# Meta-analysis of a controlled study of levosimendan combined with Sacubitril/Valsartan for the treatment of heart failure with reduced ejection fraction in China

**DOI:** 10.3389/fcvm.2024.1469457

**Published:** 2024-10-03

**Authors:** Che Li, Jifeng Zheng, Bin Zhang, Jianjiang Xu, Zhenliang Chu

**Affiliations:** ^1^ Department of Cardiology, The Second Affiliated Hospital of Jiaxing University, Jiaxing, Zhejiang, China; ^2^ Health Management Center, The Second Affiliated Hospital of Jiaxing University, Jiaxing, Zhejiang, China

**Keywords:** levosimendan, sacubitril/valsartan, reduced ejection fraction, chronic heart failure, heart function

## Abstract

**Objective:**

Levosimendan and Sacubitril/Valsartan are both potent pharmacotherapeutic agents in the clinical management of heart failure characterized by reduced ejection fraction. However, the limited efficacy of monotherapy and the lack of extensive clinical experience with combination therapy necessitate further investigation. This study aimed to evaluate the therapeutic effects of combining levosimendan with sacubitril/valsartan on chronic heart failure with reduced ejection fraction, specifically through a meta-analysis of studies conducted in China.

**Methods:**

Cochrane systematic evaluation method was used to complete data retrieval from the following related databases: (1) Wanfang database; (2) CNKI China Academic Journal Network; (3) Wipo Full-text Database of Chinese Sci-tech journals; (4) PubMed; (5) Medline; (6) Chinese Biomedical Literature Database; (7) Web of Science; and (8) Google Scholar database. We searched for studies published up to December 2021. Data were extracted from applicable articles. Meta-analyses were performed to assess the left ventricular ejection fractions (LVEF) level, NT-proBNP level, Clinical efficacy, and the left ventricular end-diastolic dimension (LVEDD) level outcomes, following PRISMA 2020 guidelines.

**Results:**

A total of five randomized controlled trials (RCTs) comprising 398 patients were included, half of the patients for levosimendan combined with Sacubitril/Valsartan and half of the patients for control groups. The effective rate in experimental group was significantly higher than that in control group [Peto-OR = 3.08, 95% CI (1.83, 5.19), *P* < 0.05]. The LVEF level after treatment in the experimental group was significantly higher than that in the control group [MD = 5.51, 95% CI (4.25, 6.76), *P* < 0.05]. After treatment, the LVEDD level in the experimental group was lower than that in the control group [MD = −3.83, 95% CI (−7.60, −0.05), *P* < 0.05]. There was a statistically significant difference in the N terminal pro B type natriuretic peptide (NT-proBNP) level between the two groups after treatment, where the value for the experimental group was lower than that for the control group [SMD = −2.68, 95% CI (−3.94, −1.43), *P* < 0.05].

**Conclusion:**

Meta-analysis results showed that levosimendan combined with Sacubitril/Valsartan has a better therapeutic effect on heart failure with reduced ejection fraction and is beneficial for improving cardiac function. The main mechanism for this may be related to the pharmacological action of levosimendan.

## Introduction

Heart failure is referred to as cardiac function failure caused by a variety of heart diseases. Its main characteristics include inadequate cardiac output, reduced tissue perfusion volume, and pulmonary circulation blood stasis (1). With changing lifestyles and population aging, in recent years, the incidence of heart failure is gradually increasing despite the continuous improvement in the related diagnosis and treatment level, and heart failure still has a high mortality rate with its three-year survival rate remains comparable to that of malignant tumors (2). How to treat heart failure effectively is a common concern for physicians. Sacubitril/Valsartan is a compound preparation, that has been widely used in the treatment of heart failure. It can effectively inhibit the over-activation of the renin-angiotensin-aldosterone system while protecting the heart function and is a first-line treatment drug recommended by the related guidelines (3). Levosimendan is a non-digitalis-positive inotropic drug, which can effectively improve the sensitivity of myocardial contractility to calcium ions and then achieve the effect of improving myocardial contractility (4, 5). Therefore, a meta-analysis of the therapeutic effect of levosimendan and Sacubitril/Valsartan on chronic heart failure patients with reduced ejection fraction was conducted to provide a theoretical basis for this combined treatment in china.

## Materials and methods

### Search strategy

The following relevant databases were searched: (1) Wanfang Database; (2) CNKI China Academic Journals Network; (3) Weipa Chinese Full-text Database of Scientific and Technological Journals; (4) PubMed; (5) Medline; (6) Chinese Biomedical Literature Database; (7) Web of Science; and (8) Google Scholar Database. The search terms included the following: (1) chronic heart failure; (2) decreased ejection fraction; (3) levosimendan; (4) Sacubitril/Valsartan. The search keywords included the following: (1) chronic heart failure; (2) decreased ejection fraction; (3) levosimendan; and (4) Sacubitril/Valsartan. Studies closely related to the above keywords were included in the analysis. Databases were searched for studies published up to December 2021. Additionally, the reference lists of review papers and every publication retrieved were manually searched for any additional published articles. Data quality assessment and relevant data extraction from all the included literature were completed by two researchers using a double-blind method of Cochrane systematic review.

### Study selection

All randomized controlled trials (RCTs) evaluating the efficacy of levosimendan and Sacubitril/Valsartan for the treatment of heart failure with reduced ejection fraction were included. Patients of chronic heart failure who had New York Heart Association (NYHA) functional classification of III or IV, received conventional anti-heart failure treatment. Patients in control group were treated with Sacubitril/Valsartan and in experimental group were treated with Sacubitril/Valsartan combined with levosimendan. The exclusion criteria were as follows: the experimental design is not rigorous; the source data are wrong and repeatedly reported; the diagnosis and efficacy judgment criteria are not standardized; use of the *in vitro* or animal experiments; and the study object data are missing.

### Data extraction

Data extraction was completed after retrieving relevant literature by two reviews independently. Each reviewer first read the titles of the articles and the abstracts, removed the reports that were inconsistent with the inclusion criteria, and then carefully read the whole text, further eliminating unsuitable manuscripts. and any disagreement was resolved by a third researcher.

The final decision about the inclusion of the manuscript was then made. The following data were extracted from each study: the first author's name, publication year, regimes for intervention and control groups, number of patients in each group, and drug duration of administration, The basic observation indicators included the following: (1) clinical efficacy (6): clinical symptoms and signs are significantly improved, left ventricular ejection fractions (LVEF) is ≥50% or increased by ≥6%, NYHA class increased by ≥2 are excellent; clinical symptoms and signs have improved, LVEF is 45%−49% or increased by 3%−5%, and NYHA class increased by 1 are effective; clinical symptoms, signs, LVEF, and NYHA class without improvement are ineffective. total effectiveness = (excellent + effective)/total number of people × 100%. (2) LVEF, left ventricular terminal diastolic diameter (LVEDD), and NT-proBNP before and after treatment.

### Quality assessment

The quality of the included studies (randomized controlled trials, RCTs) was independently evaluated by two authors (Che Li and Zhenliang Chu), and any differences were resolved through discussion and adjudication. A quality evaluation of the included literature was carried out according to the bias risk assessment criteria, these included addressing whether grouping was random, whether blinding was used, whether blinding was hidden and whether the study results data were complete. Grade A was assigned to a study when all of the above criteria were met. Grade B was assigned if some of the above criteria were met. Grade C was assigned if almost none of the above criteria were satisfied.

### Statistical analysis

The data analysis was performed with the RevMan 5.3 software, which describes the outcome indicators of categorical variables using risk ratio (RR) and its 95% confidence interval (CI). At the same time, the heterogeneity analysis was completed using the I^2^ test. No heterogeneity was present when the I^2^ was <50%. The fixed-effect and random effects models were then implemented. The test level was α=0.05.

## 2 Results

### 2.1 Basic features of the included studies

**Figure 1:**
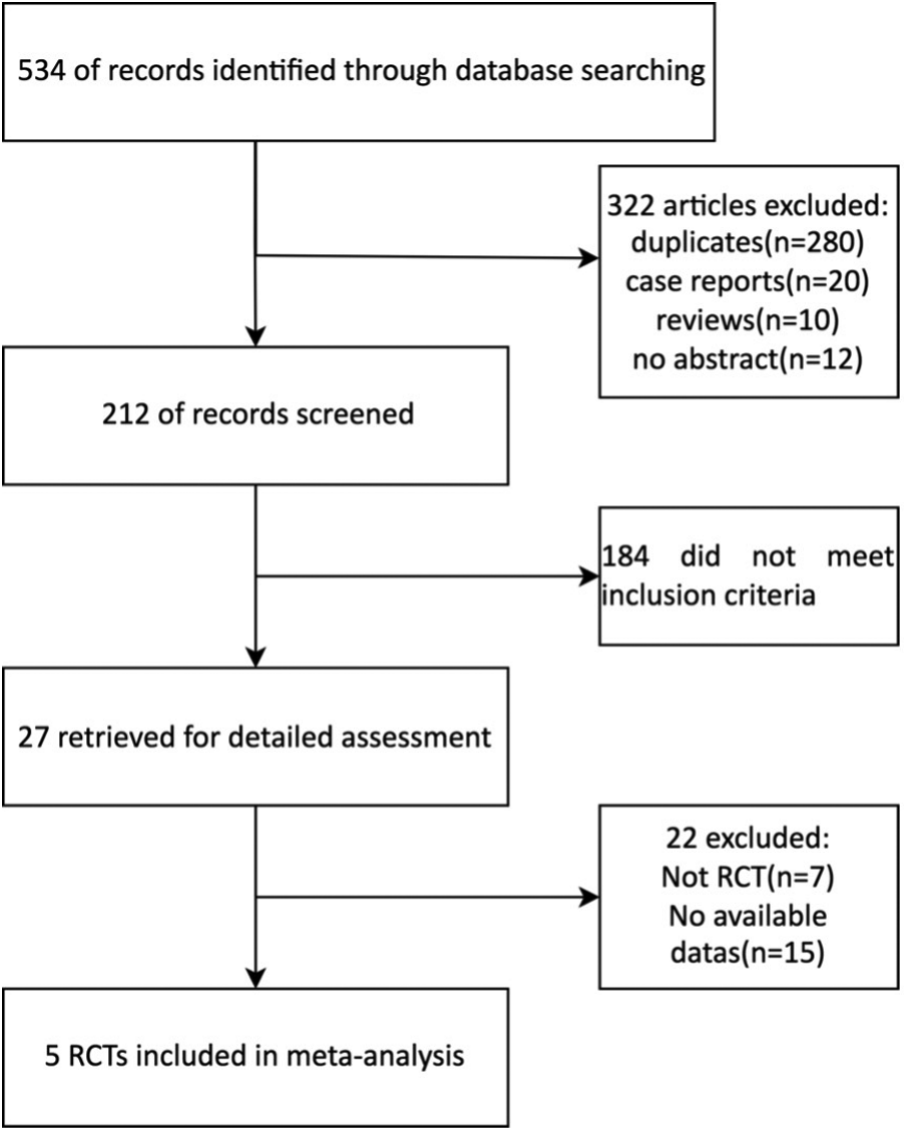
Flow diagram of the study selection process in the meta-analysis.

A total of 20 Chinese and 7 English reports were preliminarily obtained using the search keywords. After reading the questions and report contents, 15 Chinese and 7 English manuscripts did not meet the standard and were excluded. As a result, 5 articles were included in the final analysis with a total of 398 patients (Figure 1, Table 1).

**Table 1:** Basic features of the included literature.

Included studies	Number of patients		Age (years)		LVEF (%)		LVEDD (mm)		NT-proBNP (pg/ml)		Intervening measure		Time of therapy (weeks)	The basic observation indicators
	E	C	E	C	E	C	E	C	E	C	E	C		
Lu X, et al. (7)	30	30	62.30 ± 9.10	65.20 ± 7.40	27.23 ± 5.03	25.52 ± 5.01	62.03 ± 3.08	63.44 ± 5.21	7,567.43 ± 3,403.20	7,889.15 ± 4,321.34	LEV + SV	SV	4	①, ②, ③, ④
Xiong JH, et al. (8)	24	24	65.45 ± 6.08	65.17 ± 5.25	31.85 ± 4.38	31.57 ± 4.21	69.07 ± 6.88	68.58 ± 7.21	4,618.34 ± 308.27	4,627.48 ± 314.18	LEV + SV	SV	4	①, ②, ③, ④
Zheng X, et al. (9)	30	30	68.76 ± 4.94	67.83 ± 4.62	*N*	*N*	*N*	*N*	3,761.08 ± 110.24	3,764.08 ± 110.26	LEV + SV	SV	4	①, ④
Jin ZJ, et al. (10)	31	31	*N*	*N*	29.00 ± 10.00	30.00 ± 9.00	61.00 ± 7.00	60.00 ± 5.00	8,000.00 ± 1,400.00	8,000.00 ± 1,300.00	LEV + SV	SV	4	①, ②, ③, ④
Zhu QH (11)	84	84	62.20 ± 5.20	61.40 ± 4.60	34.4 ± 5.7	34.2 ± 6.0	*N*	*N*	1,576.5 ± 223.40	1,552.6 ± 213.50	LEV + SV	SV	4	①, ②, ④

E, experimental group; C, control group; *N*, not provide; LEV, levosimendan; SV, sacubitril/valsartan; LVEF, left ventricular ejection fractions; LVEDD, left ventricular end-diastolic dimension; ① clinical efficacy; ② LVEF; ③ LVEDD; ④ NT-proBNP.

### 2.2 Methodological evaluation of the quality of the included literature quality

All of the included literature were RCTs with a quality evaluation grade of B (Table 2).

**Table 2:** Summary of the quality evaluation of included trials.

Study or subgroup	Randomizing	Blinding	Hidden grouping	Data integrity	Quality evaluation grade
Lu X (7)	Not described	Yes	Not described	Yes	B
Xiong JH (8)	Not described	Yes	Not described	Yes	B
Zheng X (9)	Not described	Yes	Not described	Yes	B
Jin ZJ (10)	Not described	Yes	Not described	Yes	B
Zhu QH (11)	Not described	Yes	Not described	Yes	B

### 2.3 Contrast in total effective rate between two groups

**Figure 2:**
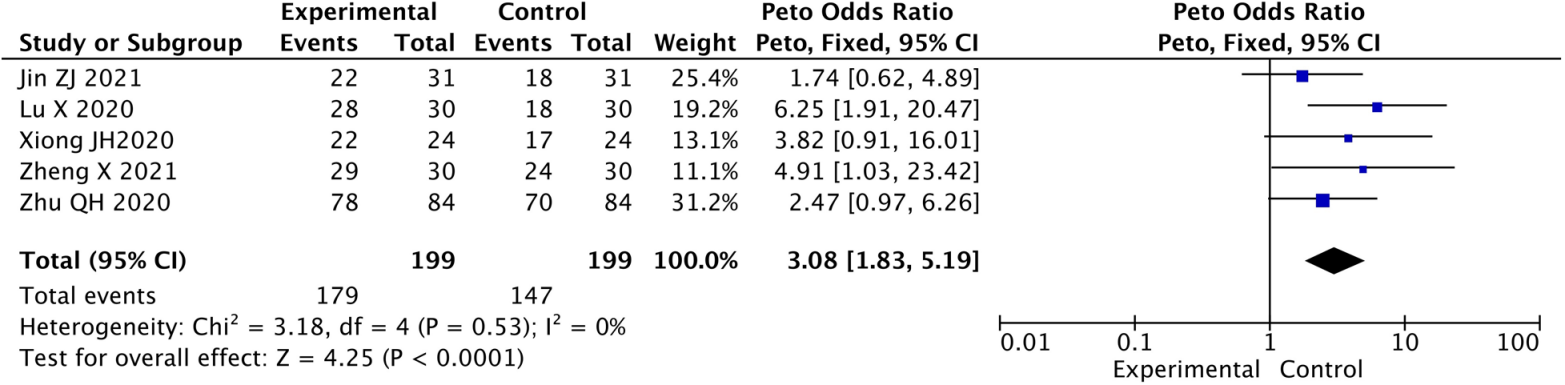
Contrast in effective rate between two groups.

The effective rate in experimental group was significant higher than that in control group [Peto-OR = 3.08, 95% CI (1.83, 5.19), *P* < 0.05] (Figure 2).

### 2.4 Contrast in LVEF level between two groups after treatment

**Figure 3:**
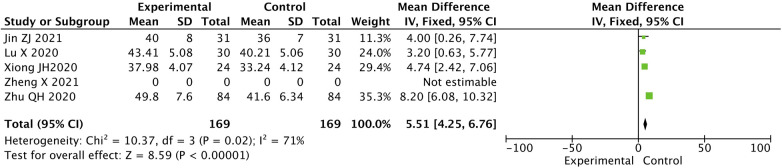
Contrast in LVEF level between two groups after treatment.

The difference in the LVEF level in the two groups after treatment was statistically significant. The value for the experimental group was higher than that for the control group [MD = 5.51, 95% CI (4.25, 6.76), *P* < 0.05] (Figure 3).

### 2.5 Contrast in LVEDD level between two groups after treatment

**Figure 4:**
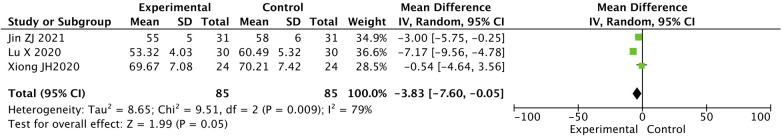
Contrast in LVEDD level between two groups after treatment.

The LVEDD level in the two groups after treatment was statistically significantly different. The value for the experimental group was lower than that for the control group [MD = −3.83, 95% CI (−7.60, −0.05), *P* < 0.05] (Figure 4).

### 2.6 Contrast in NT-proBNP level between two groups after treatment

**Figure 5:**
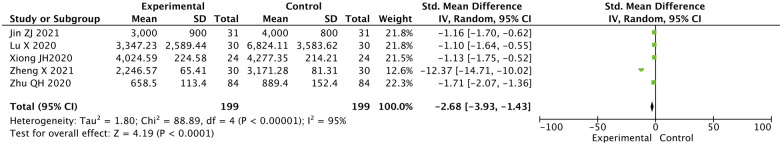
Contrast in NT-proBNP level between two groups after treatment.

The NT-proBNP level in the two groups after treatment was statistically significantly different, the value for the experimental group was lower than that for the control group [SMD = −2.68, 95% CI (−3.94, −1.43), *P* < 0.05] (Figure 5).

## 3 Discussion

Some studies have suggested that abnormal activation of the sympathetic nervous system and the endocrine system may be involved in the occurrence and development of heart failure, ultimately leading to pathological changes such as ventricular remodeling and myocardial fibrosis (12), further resulting in impaired ventricular diastolic function and increased risk of arrhythmias and sudden cardiac death (13). Increasingly, research indicates that the effectiveness and safety of sacubitril/valsartan and levosimendan are good in the treatment of heart failure patients with reduced ejection fraction (14), but there is limited experience with the combination therapy of both drugs. In this analysis, we reviewed five studies, including 398 heart failure patients with reduced ejection fraction, and conducted a meta-analysis. The results showed that the combination of levosimendan and sacubitril/valsartan was more effective in treating heart failure with reduced ejection fraction and contributed to the improvement of patients’ cardiac function.

In the 2017 American College of Cardiology/American Heart Association/American Heart Failure Society of America (ACC/AHA/HFSA) guidelines, sacubitril/valsartan was recommended as the standard therapy for heart failure patients (15). The results of this study have shown that the experimental group had a higher overall effectiveness rate compared to the control group, indicating that the combination of sacubitril/valsartan with levosimendan is more effective in treating heart failure with reduced ejection fraction. This finding is consistent with previous related research (16). The analysis suggests that using sacubitril/valsartan alone lacks effective regulation of myocardial enzymes, resulting in reduced efficacy. Levosimendan, on the other hand, can compensate for this deficiency to some extent by enhancing myocardial contractility and calcium ion sensitivity, thereby regulating myocardial enzyme levels. The combination of the two drugs has a synergistic effect, leading to improved clinical treatment outcomes. Additionally, the LVEF levels in the experimental group were higher than those in the control group, while LVEDD and serum NT-proBNP levels were lower than in the control group. This reflects that levosimendan in combination with sacubitril/valsartan can effectively enhance the cardiac function of heart failure patients with reduced ejection fraction. The reason for this is that levosimendan can effectively increase the sensitivity of myocardial cells to calcium ions, thereby enhancing myocardial contractile function without affecting the cellular calcium mechanism. This can conserve ATP function, reduce total peripheral vascular resistance, and pulmonary artery pressure, and further improve heart function. Other studies have reported that levosimendan can effectively dilate blood vessels, reduce peripheral vascular resistance, and thereby reduce the cardiac load, ultimately leading to improved heart function (17).

This study has certain limitations. For instance, it did not analyze the cardiac index and adverse reactions of patients before and after treatment, which may result in some limitations in the study findings. The study population was limited to China, and the treatment experience in countries and regions outside of China was insufficient. The sample size included in the study was relatively small, with a limited number of patients in each study. Therefore, due to the limited number of original studies, the small number of patients in each subgroup, and significant differences in study designs, the statistical power of this study's results was weakened. In future research, efforts should be made to increase the sample size and expand the analysis of relevant indicators to obtain more comprehensive and reliable data.

## Conclusion

The combination of levosimendan and sacubitril/valsartan is more effective in the treatment of heart failure with reduced ejection fraction and contributes to the improvement of patients’ cardiac function. Therefore, the clinical application of sacubitril/valsartan in combination with levosimendan is recommended for heart failure patients with reduced ejection fraction. It is imperative to undertake extensive multicenter randomized controlled trials to corroborate these meta-analysis findings, rendering them suitable for practical clinical adoption.

## Data Availability

The original contributions presented in the study are included in the article/Supplementary Material, further inquiries can be directed to the corresponding author.
